# A-to-I RNA Editing Affects lncRNAs Expression after Heat Shock

**DOI:** 10.3390/genes9120627

**Published:** 2018-12-13

**Authors:** Roni Haas, Nabeel S. Ganem, Ayya Keshet, Angela Orlov, Alla Fishman, Ayelet T. Lamm

**Affiliations:** Faculty of Biology, Technion-Israel Institute of Technology, Technion City, Haifa 32000, Israel; ronime@campus.technion.ac.il (R.H.); nabeelsg@campus.technion.ac.il (N.S.G.); ayya.keshet@gmail.com (A.K.); angelaorlov@gmail.com (A.O.); fishmana@technion.ac.il (A.F.)

**Keywords:** stress response, ADAR, *Caenorhabditis elegans*, transcriptomics

## Abstract

Adenosine to inosine (A-to-I) RNA editing is a highly conserved regulatory process carried out by adenosine-deaminases (ADARs) on double-stranded RNA (dsRNAs). Although a considerable fraction of the transcriptome is edited, the function of most editing sites is unknown. Previous studies indicate changes in A-to-I RNA editing frequencies following exposure to several stress types. However, the overall effect of stress on the expression of ADAR targets is not fully understood. Here, we performed high-throughput RNA sequencing of wild-type and ADAR mutant *Caenorhabditis elegans* worms after heat-shock to analyze the effect of heat-shock stress on the expression pattern of genes. We found that ADAR regulation following heat-shock does not directly involve heat-shock related genes. Our analysis also revealed that long non-coding RNAs (lncRNAs) and pseudogenes, which have a tendency for secondary RNA structures, are enriched among upregulated genes following heat-shock in ADAR mutant worms. The same group of genes is downregulated in ADAR mutant worms under permissive conditions, which is likely, considering that A-to-I editing protects endogenous dsRNA from RNA-interference (RNAi). Therefore, temperature increases may destabilize dsRNA structures and protect them from RNAi degradation, despite the lack of ADAR function. These findings shed new light on the dynamics of gene expression under heat-shock in relation to ADAR function.

## 1. Introduction

The survival of an organism depends on its ability to cope with environmental stresses. Exposure to elevated temperatures, oxidants, and heavy metals results in protein misfolding and aggregation, leading to protein homeostasis (proteostasis) disruptions. To counteract these detrimental effects, organisms initiate heat-shock response (HSR), an ancient mechanism, conserved from archaebacteria to mammals [1,2]. The central players of HSR are molecular chaperones called heat-shock proteins (HSPs). An increase in the level of misfolded proteins following heat shock activates a regulator of HSP transcription, heat-shock factor 1 (HSF1) [1,2], resulting in expression of HSP, which bind to misfolded proteins and enable their refolding into native conformations [2].

In multicellular organisms, HSR is not cell-autonomous, but rather regulated by neuronal and endocrine pathways. This is done to integrate the response with other organismal processes. In *Caenorhabditis *elegans,** the temperature-dependent transcription of HSP genes by HSF1 in all the somatic cells is induced by a pair of thermosensory neurons, the named AFDs, and their postsynaptic partners, named AIYs [3].

Adenosine to inosine (A-to-I) RNA editing is a highly conserved process, and is known to regulate cell function under various stress conditions [4,5]. A-to-I RNA editing is a post-transcriptional RNA modification within double-stranded RNA structures by the adenosine deaminase acting on RNA (ADAR) family of enzymes [6]. A-to-I RNA editing is common to all metazoans and is a very abundant modification of the transcriptome [7,8]. However, the biological significance of the phenomena is still not fully understood. Since inosine is read as guanosine by the translational machinery, A-to-I RNA editing can recode the transcriptome by substituting amino acids [9,10] and altering exon splice sites [11]. Recoding by editing diversifies the proteome thereby improving fitness. Editing is important to the central nervous system’s (CNS) function as the majority of recoding events in *Drosophila* and humans affect proteins involved in neurotransmission [12,13]. However, most of the editing events in human and in *C. elegans* occur in non-coding regions including introns, 3′ and 5′ untranslated regions (UTRs) and repetitive elements [8], suggesting additional roles for editing. For instance, editing destabilizes RNA duplexes by converting A-U base pairs to I-U wobbles [14]. In mammals, the disruption of secondary structures of endogenous transcripts by ADAR1 prevents activation of the cytosolic innate immune system (Interferon (IFN) response to viral infection) [14]. Similarly, in *C. elegans,* A-to-I editing protects endogenous double-stranded RNAs (dsRNAs) from RNA-interference (RNAi), a small interfering RNA (siRNA)-based innate immune mechanism aimed at destroying invading viral RNA [15,16]. Absence of adenosine-deaminases (ADARs) leads to an enrichment of siRNAs matching the hyper-edited genomic regions and to the downregulation of pseudogenes and long non-coding RNAs (lncRNAs) in an RNAi dependent manner [17,18]. Since long double-stranded RNAs are predominantly ADAR targets [19], the prominent effect of A-to-I editing on pseudogenes and lncRNAs is not surprising, due to their redundant patterns [17,20]. In contrast to mice and humans, *C. elegans* lacking ADAR genes are viable, but exhibit neurological defects (impaired chemotaxis), as well as a reduced lifespan and reduced expression of transgenes [21,22].

Editing probably only happens in the context of dsRNA. However, the RNA secondary structure is highly dynamic, as it stabilizes at low temperatures and melts at high temperatures. Therefore, in ectotherms, temperature changes result in editing efficiency changes, making A-to-I editing an optimal mechanism for sensing and regulating temperature adaptation. For example, in the octopus, the level of Kv1 potassium channel messenger RNA (mRNA) editing is much higher in the Antarctic species than those living in the tropics. The recoding by editing provides the channel with a much faster closing kinetics enabling it to maintain rapid firing rates in the cold [9].

In *Drosophila* the ADAR enzyme undergoes auto-RNA editing under certain conditions, resulting in a less efficient auto-regulatory isoform. Lowering or raising the temperature by 10 °C causes a 20% increase and 30% reduction in auto-editing levels, respectively [23]. In addition, the editing levels of sites within mRNA transcripts decrease following exposure to heat [4,24]. The decreased editing level results from alterations in the structure of ADAR’s target RNA, as well as from a decrease in the expression level of ADAR at high temperatures [25]. Examining the temperature-dependent changes of the entire editome of *Drosophila* revealed that although editing levels at elevated temperature are lower, the number of edited sites is significantly greater and they are less evolutionarily conserved [4]. These results suggest that due to changes in dsRNA structures at high temperatures, ADAR might not bind to its targets, but possibly non-specifically bind to thermodynamically stable dsRNA. On the phenotypic level, mutant flies lacking *ADAR* exhibit considerably more behavioral defects at high temperatures [26], and flies expressing low levels of ADAR protein fail to respond to temperature elevation by shifting to an adaptive nocturnal activity pattern [4]. Thus, ADAR function seems to play an important part in heat-stress response regulation.

The role of A-to-I RNA editing in the adaptation of nematodes to temperature changes has not been previously assessed. To get insight into the roles of A-to-I RNA editing in the adaptation of *C. elegans* to elevated environmental temperatures, we evaluated the overall gene expression changes under restricted heat-shock conditions (34 °C) in wild-type *C. elegans* worms and in strains mutated in both ADAR genes, *adr-1* and *adr-2*. Our results indicate that despite the regulatory role of ADAR enzymes upon heat stress, the expression of heat shock related genes is not affected in the absence of RNA editing following heat shock. In addition, a class of RNA editing target genes, lncRNAs, is upregulated under heat shock in ADAR mutants, suggesting that temperature increases may affect the function of ADAR enzymes in protecting against RNAi.

## 2. Materials and Methods

### 2.1. Maintenance and Growth of Caenorhabditis elegans Strains

The following strains were used in this study: Bristol N2 [27], BB4 *adr-1*(gv6) I; *adr-2*(gv42) III [22], and BB21 *adr-1*(tm668) I; and *adr-2*(ok735) III [28]. Only self-crossed worms were used in this study. Strains were maintained at 20 °C on Nematode Growth Medium (NGM) with OP50 bacteria for food and cultured as described in [27]. For embryo isolation, gravid N2 adults were treated with sodium hypochlorite solution to dissolve animals of all stages except embryos. The embryos were incubated in M9 media at 20 °C for 24 h with no food supply. Hatched synchronized L1 larva were grown at 20 °C until they reached the L4 larval stage.

### 2.2. Heat Shock Treatment

For heat-shock treatment, L4 larva were transferred from the 20 °C incubator to a 34 °C water bath for 75 min incubation. Next, the worms were allowed to recover at room-temperature for 20 min and then returned to the 20 °C incubator for an additional 3 h. Control plates were kept at 20 °C for the entire time. Three biological replicas for N2 and five biological replicas for ADAR mutants (three and two for BB4 and BB21, respectively), were used to perform the heat shock experiments. Before collecting the worms, the heat-exposed and the control worms were washed several times with M9 media to avoid contamination with bacteria, and then snap-frozen in liquid nitrogen and stored at −80 °C.

### 2.3. RNA Extraction and mRNA Library Preparation

The frozen worms were ground to powder using pre-chilled mortars and pestles. RNA was extracted using Direct-Zol RNA MiniPrep Plus (Zymo research, Irvine, CA, USA) or mirVana (Ambion, Austin, TX, USA) according to the manufacturer’s instructions. RNA concentrations were measured by a Qubit^®^ Fluorometer using the Qubit^®^ RNA HS Assay Kit (Thermo Fisher Scientific, Waltham, MA, USA) and its quality was confirmed by TapeStation (Agilent Technologies, Santa Clara, CA, USA). Poly(A) enriched libraries were prepared from RNA samples using the Illumina TruSeq^®^ RNA Sample Preparation kit (Illumina, San Diego, CA, USA).

### 2.4. RNA-Seq and Gene Expression Analysis

Messenger RNA sequencing by Illumina HiSeq 2500 technology was performed with single reads of 50 bp. Sequence quality was confirmed using FastQC [29]. Reads were trimmed, collapsed and aligned to the WS220 gene transcripts of the *C. elegans* [30], using Bowtie [31], allowing multiple alignments to include multiple transcripts due to alternative splicing. The expression levels for each gene were evaluated by read counts. For gene expression analysis, the two mutant strains BB4 and BB21 were analyzed together as “ADAR mutant” to avoid allele specific changes. We validated that the gene expression levels of the two ADAR mutant strains are highly correlated (Figure S1). The DESeq2 package [32] in R was used to identify differentially-expressed genes upon heat shock for each strain, while all samples were taken into consideration in the DEseq2 design. The DESeq2 package includes automatic strict filtering of low normalized gene counts to increase power and to ensure reliable results [32]. To declare significance for genes that were differentially expressed under heat shock, we chose a *p*-value threshold after False Discovery Rate (FDR) correction of 0.05. Out of all significant genes, we considered only genes that had |log2FoldChange| >2 for the analysis. We considered differentially-expressed genes that are specific for one of the strains if in the other strain the gene had a *p*-value >0.1 and |log2FoldChange| < 2. To test the difference in the expression pattern of 3’UTR edited genes and pseudogenes compared to all genes, the DEseq [33] package in R was used to identify differentially-expressed genes and a Welch two-sample *T*-test was performed on only transcripts with *p*-adjusted ≤0.05. The lists of 3’UTR edited genes and pseudogenes that were used for the analysis are as in [17].

### 2.5. Data Availability

The sequence data from this study was submitted to the NCBI Gene Expression Omnibus (http://www.ncbi.nlm.nih.gov/geo/), accession number GSE122015.

## 3. Results

### 3.1. Gene Expression Under Standard Conditions and upon Heat-Shock

**Figure 1:**
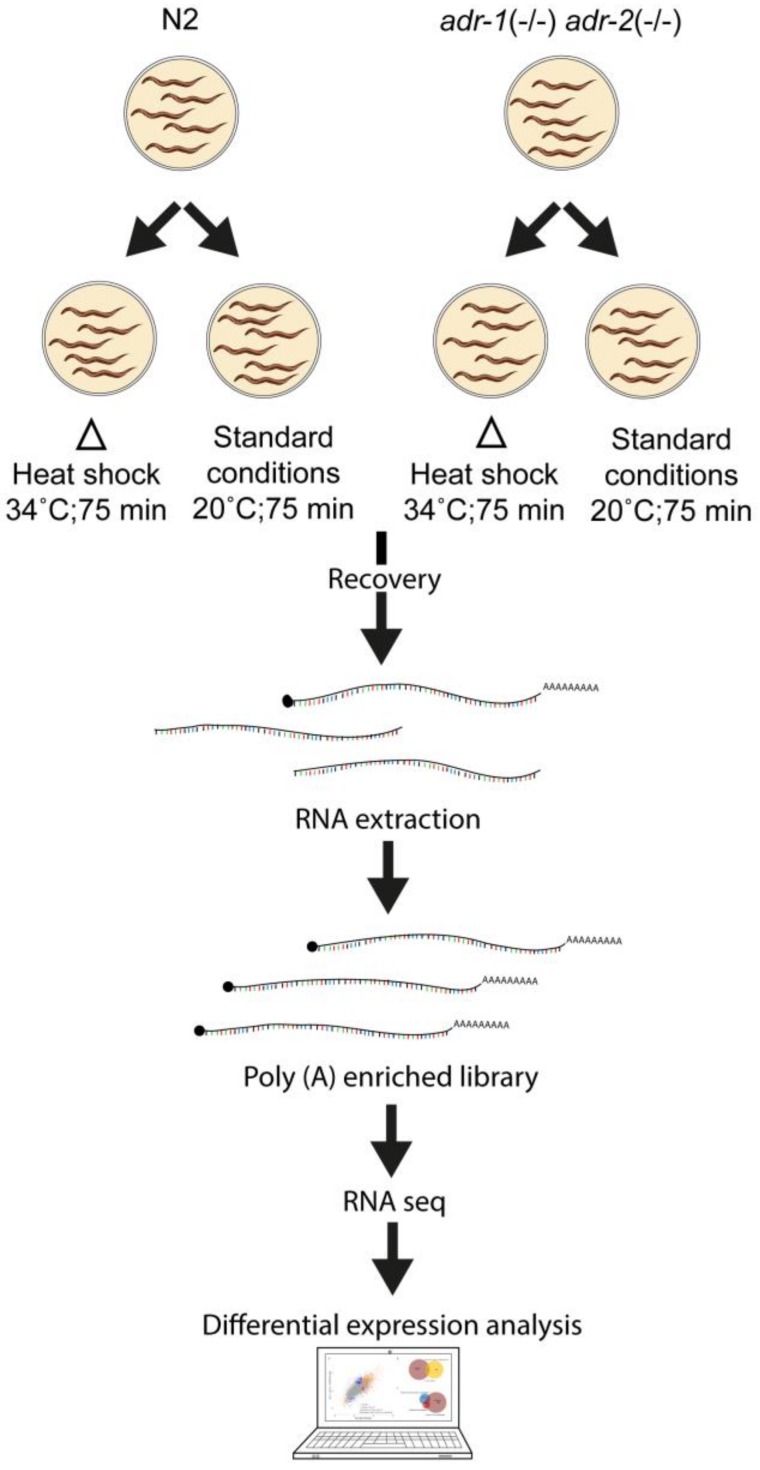
Schematic view of the heat-shock experiments.

To investigate the overall effect of stress on the expression pattern of ADAR targets, we heat stressed (34 °C) three and five biological replicas of N2 wild-type and ADAR mutant worms, respectively, at the L4 stage. For ADAR mutants, we used two different strains that contain distinct alleles (BB21 *adr-1*(tm668) I; *adr-2*(ok735) III and BB4 *adr-1*(gv6) I; and *adr-2*(gv42) III) to make sure that the heat shock effect is not allele specific. In parallel, we exposed the same strains to standard conditions (20 °C) as controls. After recovery, poly(A) enriched libraries were generated from each sample and high-throughput mRNA-sequencing (mRNA-seq) was carried out to directly evaluate the gene expression pattern of the different strains after heat shock (see experimental flow scheme, Figure 1).

**Figure 2:**
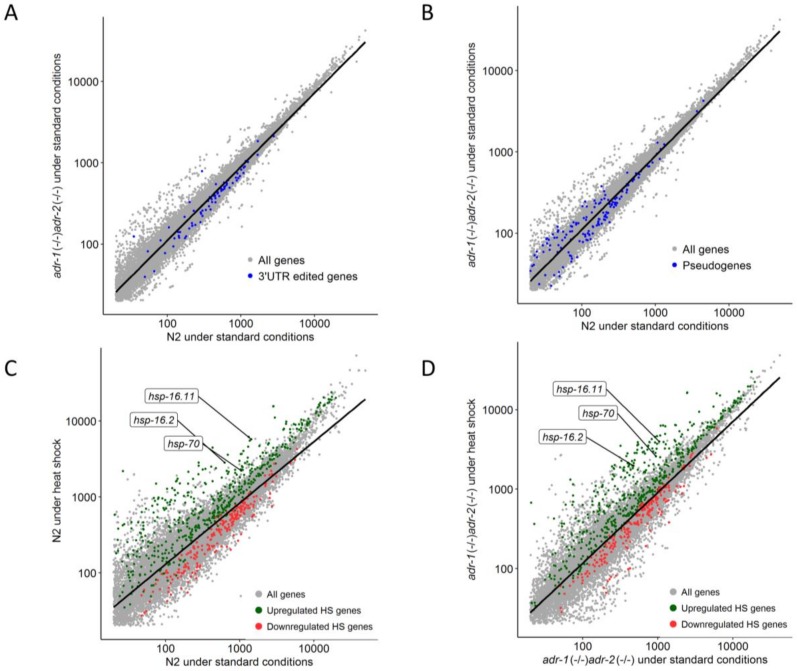
Gene expression changes under standard conditions and upon heat shock. (**A**,**B**) Log scale plots presenting normalized gene counts of at least three biological samples in adenosine-deaminases (ADAR) mutant worms compared to wild-type (N2) worms, under standard conditions. Grey dots represent all genes (n = 12,059), blue dots represent 3′ UTR-edited genes (n = 81) (**A**) and pseudogenes and long non-coding RNAs (lncRNAs) (n = 216) (**B**), and the black line is the regression line for all genes. (**C**,**D**) Log scale plots representing normalized gene counts of at least three biological samples under heat shock compared to standard conditions, for N2 (**C**) and for ADAR mutant (**D**). Grey dots are all genes, the black line is the regression line for all genes, green and red dots are upregulated and downregulated heat shock related genes, respectively, as reported by Brunquell, et al. [34] that had the same tendency in our study. HS: heat shock. For all figures (**A**–**D**), only genes that their normalized gene count was higher than 20 are presented.

We initially tested the changes in gene expression between the wild-type and ADAR mutant strains under standard conditions. Consistent with our previous data for L4 developmental stage [17], the expression of 3’UTR edited genes was reduced in relation to all genes in ADAR mutant worms compared to the wild-type (*p*-value = 3.4 × 10^−7^, Welch two-sample *T*-test) (Figure 2A) and in contrast, the expression of pseudogenes and lncRNAs was slightly increased (*p*-value = 7.7 × 10^−3^, Welch two-sample *T*-test) (Figure 2B). The changes were subtle when examining individual genes. We further examined the differences in gene expression following heat shock in each strain (Supplementary Material File S1) and tested whether genes previously identified as related to heat-shock [34] had the same tendency of upregulation or downregulation in our study as a response to the stress. Indeed, a large group of known heat-shock related genes was upregulated or downregulated, both for the wild-type and the ADAR mutants (Figure 2C,D, Supplementary Material File S2) as found earlier [34] (note that some of the genes were not significantly altered but had the expected tendency). The genes *hsp-16.2*, *hsp-16.11*, and *hsp-70,* which are considered as hallmarks of the heat-shock response [34], were specifically examined. Indeed, these genes were upregulated in both strains after heat shock (Figure 2C,D). Therefore, our experiment resulted in an effective heat-shock response and known heat-shock related genes were differentially expressed upon our heat-shock conditions, with and without A-to-I RNA editing.

### 3.2. Expression of Heat-Shock Genes upon Heat Stress Is Not Affected by Adenosine-Deaminases (ADAR) Function

**Figure 3:**
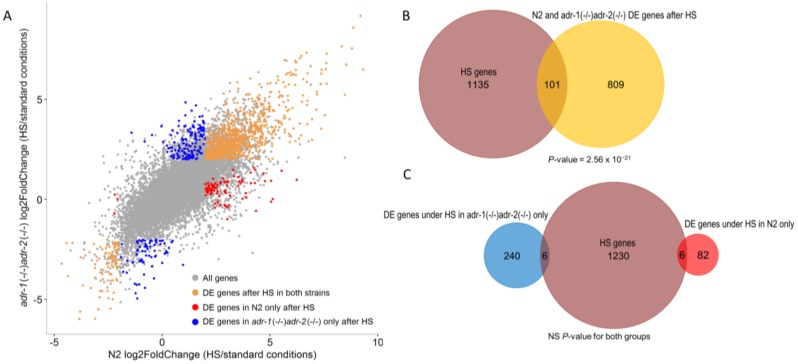
Differentially-expressed sets of genes under heat shock that are specific to the ADAR mutant or N2 and are not heat-shock related genes. (**A**) A plot representing the Log2 fold-change values (gene expression after heat shock versus standard conditions) of the ADAR mutants vs. the wild-type (N2). Grey dots represent all genes, orange dots represent significant differentially-expressed genes in both ADAR mutant and N2 worms, blue dots represent significant differentially-expressed genes specific to ADAR mutant worms, and red dots represent significant differentially-expressed genes specific to N2 worms. DE: differentially expressed. (**B**) A Venn diagram showing that the previously identified heat-shock related genes are highly enriched among genes that were differentially expressed in both ADAR mutants and N2. The number of heat shock genes and the differentially-expressed genes are shown in brown and orange, respectively. (**C**) A Venn diagram depicting that previously identified heat-shock genes are not enriched among genes that were only differentially expressed in either ADAR mutant or N2. The number of heat-shock genes, differentially-expressed genes in the ADAR mutants only, and differentially-expressed genes in N2 only are shown in brown, blue and red, respectively. NS: non-significant (*p*-value > 0.05).

To analyze the effect of heat shock stress on the expression pattern of edited genes, and genes that are indirectly regulated by A-to-I RNA, we compared between the ADAR mutants and the wild-type gene-expression changes upon heat shock. Only genes with a |log2FoldChange| > 2 and a *p*-value threshold after FDR correction of 0.05 were considered significantly differentially-expressed genes. The results indicate the existence of genes that are similarly affected upon heat shock in both the wild-type and the ADAR mutants, but also of a group of genes that is variously expressed in only one of the strains (Figure 3A). The number of genes that were only differentially expressed in the ADAR mutants was 2.8-fold higher than in the wild-type.

Most of the genes whose expression was altered under heat shock were upregulated (81% and 93% for the ADAR mutants and the wild-type, respectively) with a small fraction of downregulated genes (Supplementary Material File S1). If considering the genes that were differentially-expressed only in the wild-type, 97% were upregulated. However, interestingly, among genes that were differentially-expressed only in ADAR mutants, just 72% were upregulated, which is less than expected by chance compared to the 81% upregulation among all genes whose expression was altered (*p*-value = 4.9 × 10^−5^, hypergeometric test). Therefore, ADAR’s function under heat shock may be to prevent the downregulation of genes, possibly due to their importance for cell function under stress conditions.

To examine whether RNA editing regulation under heat stress directly involves heat-shock genes, we tested the number of the genes that were significantly differentially expressed upon heat shock in this study that are classified as known heat-shock related genes. We found that the heat-shock related genes were highly enriched in the group of genes that were significantly differentially-expressed in both the wild-type and the ADAR mutants strains (Figure 3B, *p*-value = 2.56 × 10^−21^, hypergeometric test). However, heat-shock related genes were not enriched in the significant differentially-expressed sets of genes that are specific to the wild-type or to the ADAR mutants under heat shock (Figure 3C, *p*-value > 0.05). We concluded that A-to-I RNA editing plays a part in the regulation of heat stress, but does not directly affect the expression of heat-shock related genes.

### 3.3. Pseudogenes and lncRNAs Are Differentially Expressed upon Heat Shock in Adenosine-Deaminases (ADAR) Mutants

**Figure 4:**
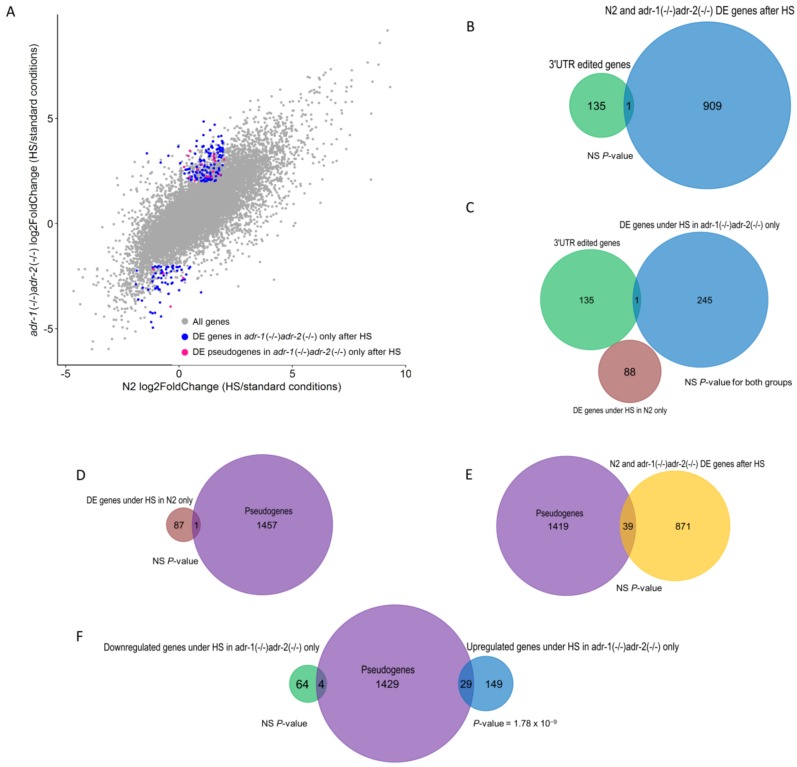
Long non-coding RNAs and pseudogenes are upregulated upon heat shock in ADAR mutants. (**A**) Scatter plot presenting log2 fold-change values (gene expression after heat shock vs. standard conditions) of the ADAR mutants compared to the wild-type (N2). Grey dots are all genes, blue dots are significant differentially-expressed genes specific to ADAR mutants, purple dots are significantly differentially-expressed pseudogenes specific to ADAR mutants. (**B**,**C**) Venn diagrams showing that genes edited at their 3’UTR are not enriched among genes that were differentially expressed under heat shock. The number of 3’UTR edited genes, differentially expressed in both N2 and the ADAR mutants, differentially expressed in the wild-type only, and differentially expressed in the ADAR mutants only are shown in purple, orange, red, and blue, respectively. (**D**,**E**) Venn diagrams showing that pseudogenes and lncRNAs are not enriched among genes that were differentially expressed in both ADAR mutants and wild-type or in wild-type only. The number of pseudogenes, differentially-expressed genes in N2 only (**D**), and differentially-expressed genes in both N2 and ADAR mutants (**E**), are shown in purple, red, and orange, respectively. (**F**) Venn diagram depicting that pseudogenes are significantly enriched among upregulated genes, which are specific to the ADAR mutant. The number of pseudogenes, the upregulated genes, and the downregulated genes are shown in purple, blue, and green, respectively. (*p*-value > 0.05).

We previously found that the expression of 3’UTR edited genes, pseudogenes and lncRNAs is affected by RNA editing, under standard conditions [17]. Therefore, we tested whether the expression of 3’UTR-edited genes, lncRNAs, and pseudogenes is specifically affected in ADAR mutants under heat stress compared to the wild-type. Genes edited at their 3′UTR were not present among affected genes upon heat shock (Figure 4B,C). However, lncRNAs and pseudogenes were significantly enriched in upregulated genes which are specific to the ADAR mutant (Figure 4A; Figure 4F, *p*-value = 1.78 × 10^−9^, hypergeometric test), but not in the wild-type or in genes that were differentially expressed in both strains (Figure 4D,E, *p*-value > 0.05). Since we restricted the conditions to define differentially-expressed genes for significant genes that also have |log2FoldChange| > 2 (fold change > 4), the enrichment of lncRNAs and pseudogenes in upregulated genes which are specific to the ADAR mutant appears to be strong (Figure 4F). As previously mentioned, the expression of all pseudogenes and lncRNAs was slightly increased in relation to the entire genes in ADAR mutant worms compared to the wild-type, under standard conditions (Figure 2B). However, interestingly, the group of lncRNAs and pseudogenes that was enriched in upregulated genes which are specific to the ADAR mutant under heat-shock, was downregulated in ADAR mutant worms compared to the wild-type under standard conditions (Figure S2) (*p*-value = 6.7 × 10^−5^, Welch two-sample *T*-test). A possible role for A-to-I editing is to protect dsRNAs from RNAi degradation by preventing DICER from processing the dsRNAs [16,17]. Therefore, in the absence of ADARs, RNAi can process the dsRNA and cause downregulation of pseudogenes which are normally edited. Indeed, downregulation of pseudogenes was observed in our previous study, in embryos [17]. Downregulation in the current study was also seen for lncRNAs and pseudogenes that were enriched in upregulated genes specific to the ADAR mutant under heat-shock. These results suggest that temperature increases may destabilize dsRNA structures and protect them from RNAi degradation, despite the lack of ADAR function.

### 3.4. No Substantial Enrichment of Gene OntologyTerms in Adenosine-Deaminases (ADAR) Specifically Expressed Genes after Heat Shock

To see whether there is an enrichment of biological processes and molecular functions in the different groups of differentially-expressed genes, we used Gorilla by hypergeometric distribution analysis [35,36]. For the background set of genes, we used all expressed genes. The analysis resulted in substantial gene ontology (GO) terms for genes that were differentially expressed in both strains (Table S1). The top ranked enriched GO terms of biological process belong to the stress and unfolded protein response, which are classic heat-shock response mechanisms (Table S1). GO terms of molecular function were mostly related to structural activity (Table S2).

Genes that were differentially-expressed specifically in one of the strains after heat shock did not yield any significant enrichment. Since we previously restricted the conditions for genes that were significantly differentially expressed, but also had |log2FoldChange| > 2, for enrichment purposes we extended the list of genes to include all significant genes (*p*-adjusted ≤ 0.05), with no conditions regarding the log2FoldChange. This is assuming that not all genes sharing biological processes or functions are necessarily among the highly influenced genes.

For differentially-expressed genes that are specific to the ADAR mutants, significant enrichment of biological processes was still not found and only few molecular function categories were enriched. This is probably because the functions of most of the lncRNAs and pseudogenes are not annotated. However, we did find a substantial enrichment for genes that were differentially expressed only in the wild-type according to biological process (Table S1) and molecular function GO terms (Table S2). The enriched GO terms for only the wild-type are not directly related to known stress response mechanisms, as obtained for GO terms that were differentially expressed for both strains.

To confirm the enrichment, we ran the same process 10 times with randomly selected genes. Random lists did not result in enrichment of any process or function with *p*-adjusted < 0.01. Thus, the results appear to be strong.

We concluded that similarly affected genes in both the wild-type and ADAR mutants are related to stress, and genes affected in wild-type worms only are not stress related. Since the main group of affected genes in the ADAR mutant worms, are lncRNAs and pseudogenes, is not annotated, no substantial enrichment of GO terms would be obtained.

## 4. Discussion

In this study, we sought to gain insight into the roles of A-to-I RNA editing in *C. elegans* adaptation to heat stress. The ADAR mutant worms that were used contained distinct alleles, to negate any allele specific effect. Heat shock treatment was conducted under 34 °C for 75 min, which is strong enough to invoke a massive stress response but is also tolerated by the nematodes [37]. We then explored the role of RNA A-to-I editing, by comparing between the gene-expression profiles of the wild-type strain and the ADAR mutant, under heat shock. The rationale was that genes whose expression is altered in a similar manner upon heat shock are part of stress response mechanisms not affected by ADAR function. However, the A-to-I RNA editing effect can be tracked by identifying genes that are differentially altered in only one of the strains.

### 4.1. Adenosine to Inosine RNA Editing Does Not Directly Affect Heat Shock Related Genes upon Heat Stress

Since the HSR is a well-defined mechanism, we first tested the extent of changes in expression upon heat stress for known heat-shock genes and transcripts that were previously identified as related to heat-shock [34]. As expected, a large number of heat-shock related genes were upregulated or downregulated in according to previous reports in the wild-type [34]. When analyzing the number of known heat-shock related genes that were differentially expressed in only one of the strains, we did not find an enrichment. However, heat shock genes were highly enriched among the group of genes that were differentially expressed in both strains (Figure 3). These results imply that A-to-I RNA editing does not directly involve heat shock related genes as part of an adaptation to heat shock. The editing in *Drosophila* was shown to be much less efficient in elevating temperatures [23]. It is, therefore, likely that the most important group of proteins under heat shock, HSPs and their related proteins, would not be regulated by its function. It should be noted that in this study, ADAR enzymes were slightly downregulated under heat shock, but this downregulation did not reach statistical significance (Supplementary Material File S1). In addition, since in *C. elegans*, no significant examples of editing sites in coding regions that can change protein structure were found [17,19] and the changes in the expression of edited genes is mild [17], it is probable that heat-shock proteins will not be affected by the ADAR function in *C. elegans*. Corroborating these findings is the enrichment of GO terms for differentially-expressed genes in both strains that belong to response to stress and unfolded protein response, which are classic HSP response mechanisms.

It is still possible that there is some effect on the heat shock related genes, as it has been shown that the melting of mRNA secondary structures at high temperatures, directly causes the synthesis and binding of HSP transcription factors [38].

### 4.2. Adenosine to Inosine RNA Editing Regulates the Heat Stress Response

Although it was found that A-to-I RNA editing does not regulate heat-shock related genes upon heat shock, ADAR does seem to play an important part in the regulation of the heat stress response according to previous studies in *Drosophila*, mostly in behavioral patterns [4,26]. Our results suggest that ADARs in *C. elegans* counter downregulation of some of the genes under heat shock. This was shown by the significant higher fraction of downregulated genes among differentially-expressed genes that are specific to the ADAR mutants (28%) as compared to all genes whose expression levels were altered in the ADAR mutant (19%). Downregulation was minor (3%) among differentially-expressed genes that are specific to wild-type worms (Supplementary Material File S1). This may indicate that A-to-I editing protects endogenous dsRNA that stays stable under heat shock from RNAi, as happens under standard conditions [15,16]. This finding is supported by a reported molecular model suggesting that although the ADAR protein is present at lower concentrations under heat stress compared to standard conditions, highly stable RNA structures are still edited [25].

ADAR may also function to restrict the regulation of genes under stress. This is implied by the greater number of genes (2.8-fold) that are specifically differentially-expressed in the ADAR mutants compared to the wild-type. Furthermore, no substantial enrichment of GO terms was found in ADAR mutants specifically differentially expressed genes after heat shock, even when relaxing the conditions and extending the list of genes. However, we did identify distinct biological processes and molecular functions that were specific to the wild-type. Many of these biological processes and functions are related to ongoing maintenance mechanisms of the cell. It is possible that A-to-I RNA editing under heat stress is important for ensuring that the essential processes during stress do not fail. However, A-to-I RNA editing does not regulate special proteins that are evoked during heat stress.

Since the nature of A-to-I RNA editing is to be oppositely regulated under cold-shock and heat-shock, it is reasonable that its role will differ between these stress types [23]. This was seen in octopus where high editing rates provide the potassium channel with a much faster closing kinetics only in the cold, but not under high temperatures [9]. Future studies will be needed to characterize the role of ADARs under cold-shock in *C. elegans* and to compare it to heat-stress.

### 4.3. Elevating Temperatures May Destabilize dsRNA Structures and Protect Them from RNAi Degradation, Despite the Lack of Adenosine-Deaminases (ADAR) Function

Since one of the main goals of this study was to test the effect of heat-shock stress on the expression pattern of edited genes, an obvious question was whether the expression of ADAR targeted genes changes under heat-shock. We previously showed that two main groups of ADAR-target genes, lncRNAs and 3′UTR edited genes, are affected in the absence of ADARs under standard conditions [17]. We also observed the same effect under standard conditions in the current study. When testing the effect of ADAR’s absence upon heat shock on these group of genes, we found that 3’UTR edited genes were not differentially expressed upon heat shock in any of the strains. However, lncRNAs and pseudogenes were significantly upregulated upon heat shock in the ADAR mutants, but not in the wild-type or in genes that were differentially expressed in all strains. The expression of lncRNAs and pseudogenes slightly increases in ADAR mutant worms compared to the wild-type under standard conditions. However, when testing only the lncRNAs and pseudogenes that were significantly upregulated upon heat shock in the ADAR mutants, downregulation is observed under standard conditions. We have also shown that, in embryos, there is a downregulation of lncRNAs rather than upregulation [17]. This observation may indicate that although we started the heat shock treatment with L4 larval stage worms, some of the worms possibly progressed through their life cycle and generated embryos, due to ongoing incubation times subsequent to the heat shock treatment.

One suggested role for A-to-I editing is to protect dsRNA from RNAi degradation by preventing DICER from processing the dsRNA [16,17]. Therefore, under standard conditions in the absence of ADARs, RNAi processes dsRNA and causes downregulation of pseudogenes which are normally edited. The absence of this downregulation when both RNAi and ADAR enzymes are absent further confirms this theory [17]. It is possible that a temperature increase reduces the stability of the dsRNA structure, making them less desirable as DICER substrates and, therefore, even though A-to-I RNA editing is lacking, pseudogenes will be protected from RNAi degradation under heat stress conditions. For this reason, it is feasible that pseudogenes will be mostly upregulated in comparison to standard conditions in ADAR mutant strains. It is possible that the dsRNA structures of 3′UTR edited genes are more stable in elevated temperatures than the pseudogene structures and, therefore, we did not observe a change in their expression levels after heat shock in the absence of ADARs. In the wild-type, no significant change in the lncRNAs and pseudogene gene expression was detected under heat shock conditions compared to standard conditions. This is probably the result of reduced RNAi processing of the dsRNA, under both conditions. Under standard conditions, A-to-I editing protects endogenous dsRNA from RNAi and under heat-shock conditions, editing levels are decreased, but RNAi degradation is likely to be reduced as well, due to destabilized dsRNA structures. Therefore, we assume that since RNAi degradation is reduced in both conditions, significant changes in gene expression levels between heat shock and control conditions are only seen in the ADAR mutant and not in the wild-type. Further studies should test the hypothesis that increased temperature reduces RNAi degradation by destabilizing dsRNA structures. In addition, we cannot determine from our analysis whether A-to-I editing events are directly responsible for the transcriptomic changes of pseudogenes and lncRNA, as there is a possibility that other editing-independent functions are the cause for the differences. However, the naturally redundant patterns of pseudogenes and lncRNA make them desirable targets for ADARs, implying that RNA editing events directly influence pseudogenes and lncRNA expression. This assumption is corroborated by a study in mammals, showing that RNA editing events stabilize the double stranded structure of *Alu* repeats [39].

In this study we identified upregulation of lncRNA and pseudogenes in ADAR mutants, under heat stress. Upregulation of a highly edited lncRNA, rncs-1 was also seen under starvation conditions [20]. This gene was not among the genes that were significantly upregulated upon heat shock in the ADAR mutants in this study. More studies will be needed to define possible relations between editing and the upregulation of lncRNAs under various stress conditions.

To conclude, our results suggest that under standard conditions in the absence of RNA editing, RNAi processing of the dsRNA is enhanced leading to degradation and downregulation, while under heat shock the dsRNA structures are destabilized preventing RNAi processing even in the absence of RNA editing, which leads to upregulation of gene expression in ADAR mutants.

## Supplementary Materials

The following are available online at http://www.mdpi.com/2073-4425/9/12/627/s1, **Figure S1.** Gene expression levels of the BB4 and BB21 strains are highly correlated. **Figure S2.** Pseudogenes that are upregulated in the ADAR mutants under heat shock are downregulated in ADAR mutants under standard conditions. **Table S1.** Enrichment of differentially-expressed genes according to biological process GO term categories. **Table S2.** Enrichment of differentially-expressed genes according to molecular function GO term categories. **Supplementary Material File S1.** Gene expression analysis. **Supplementary Material File S2.** Heat-shock-related genes that that were upregulated or downregulated in both wild-type and ADAR mutants.

## References

[1] Morimoto R.I. (1998). Regulation of the heat shock transcriptional response: Cross talk between a family of heat shock factors, molecular chaperones, and negative regulators. Genes Dev..

[2] Lindquist A., Craig E.A. (1988). The heat-shock proteins. Annu. Rev. Genet..

[3] Prahlad V., Cornelius T., Morimoto R.I. (2008). Regulation of the cellular heat shock response in *Caenorhabditis elegans* by thermosensory neurons. Science.

[4] Buchumenski I., Bartok O., Ashwal-Fluss R., Pandey V., Porath H.T., Levanon E.Y., Kadener S. (2017). Dynamic hyper-editing underlies temperature adaptation in *Drosophila*. PLOS Genet..

[5] Garrett S.C., Rosenthal J.J.C. (2012). A Role for A-to-I RNA editing in temperature adaptation. Physiology.

[6] Savva Y.A., Rieder L.E., Reenan R.A. (2012). The ADAR protein family. Genome Boil..

[7] Nishikura K. (2015). A-to-I editing of coding and non-coding RNAs by ADARs. Nat. Rev. Mol. Cell Boil..

[8] Bazak L., Haviv A., Barak M., Jacob-Hirsch J., Deng P., Zhang R., Isaacs F.J., Rechavi G., Li J.B., Eisenberg E. (2014). A-to-I RNA editing occurs at over a hundred million genomic sites, located in a majority of human genes. Genome Res..

[9] Garrett S., Rosenthal J.J. (2012). RNA editing underlies temperature adaptation in K+ channels from polar octopuses. Science.

[10] Higuchi M., Maas S., Single F.N., Hartner J., Rozov A., Burnashev N., Feldmeyer D., Sprengel R., Seeburg P.H. (2000). Point mutation in an AMPA receptor gene rescues lethality in mice deficient in the RNA-editing enzyme ADAR2. Nature.

[11] Rueter S.M., Dawson T.R., Emeson R.B. (1999). Regulation of alternative splicing by RNA editing. Nature.

[12] Schmauss C., Howe J.R. (2002). RNA editing of neurotransmitter receptors in the mammalian brain. Sci. STKE.

[13] Hoopengardner B., Bhalla T., Staber C., Reenan R. (2003). Nervous system targets of RNA editing identified by comparative genomics. Science.

[14] Liddicoat B.J., Piskol R., Chalk A.M., Ramaswami G., Higuchi M., Hartner J.C., Li J.B., Seeburg P.H., Walkley C.R. (2015). RNA editing by ADAR1 prevents MDA5 sensing of endogenous dsRNA as nonself. Science.

[15] Ganem N.S., Lamm A.T. (2017). A-to-I RNA editing—Thinking beyond the single nucleotide. RNA Biol..

[16] Reich D.P., Tyc K.M., Bass B.L.C. (2018). *C. elegans* ADARs antagonize silencing of cellular dsRNAs by the antiviral RNAi pathway. Genes Dev..

[17] Goldstein B., Agranat-Tamir L., Light D., Ben-Naim Zgayer O., Fishman A., Lamm A.T. (2017). A-to-I RNA editing promotes developmental stage-specific gene and lncRNA expression. Genome Res..

[18] Wu D., Lamm A.T., Fire A.Z. (2011). Competition between ADAR and RNAi pathways for an extensive class of RNA targets. Nat. Struct. Mol. Biol..

[19] Zhao H.Q., Zhang P., Gao H., He X., Dou Y., Huang A.Y., Liu X.M., Ye A.Y., Dong M.Q., Wei L. (2015). Profiling the RNA editomes of wild-type *C. elegans* and ADAR mutants. Genome Res..

[20] Whipple J.M., Youssef O.A., Aruscavage P.J., Nix D.A., Hong C., Johnson W.E., Bass B.L. (2015). Genome-wide profiling of the *C. elegans* dsRNAome. RNA.

[21] Sebastiani P., Montano M., Puca A., Solovieff N., Kojima T., Wang M.C., Melista E., Meltzer M., Fischer S.E., Andersen S. (2009). RNA editing genes associated with extreme old age in humans and with lifespan in *C. elegans*. PLoS ONE.

[22] Tonkin L.A., Saccomanno L., Morse D.P., Brodigan T., Krause M., Bass B.L. (2002). RNA editing by ADARs is important for normal behavior in *Caenorhabditis elegans*. EMBO J..

[23] Savva Y.A., Jepson J.E.C., Sahin A., Sugden A.U., Dorsky J.S., Alpert L., Lawrence C., Reenan R.A. (2012). Auto-regulatory RNA editing fine-tunes mRNA re-coding and complex behaviour in *Drosophila*. Nat. Commun..

[24] Stocker J., Huang H.W., Wang H.M., Chang H.W., Chiu C.C., Cho C.L., Tseng C.N. (2013). Reduction of RNA A-to-I editing in *Drosophila* acclimated to heat shock. Kaohsiung J. Med. Sci..

[25] Rieder L.E., Savva Y.A., Reyna M.A., Chang Y.J., Dorsky J.S., Rezaei A., Reenan R.A. (2015). Dynamic response of RNA editing to temperature in *Drosophila*. BMC Biol..

[26] Palladino M.J., Keegan L.P., O’Connell M.A., Reenan R.A. (2000). A-to-I Pre-mRNA Editing in *Drosophila* is primarily involved in adult nervous system function and integrity. Cell.

[27] Brenner S. (1974). The genetics of *Caenorhabditis elegans*. Genetics.

[28] Hundley H.A., Krauchuk A.A., Bass B.L.C. (2008). *C. elegans* and *H. sapiens* mRNAs with edited 3’ UTRs are present on polysomes. RNA.

[29] Koudande O.D., Iraqi F., Thomson P.C., Teale A.J., van Arendonk J.A. (2000). Strategies to optimize marker-assisted introgression of multiple unlinked QTL. Mamm. Genome Off. J. Int. Mamm. Genome Soc..

[30] WormBase. http://www.wormbase.org.

[31] Langmead B. (2010). Aligning short sequencing reads with Bowtie. Curr. Protoc. Bioinform..

[32] Love M.I., Huber W., Anders S. (2014). Moderated estimation of fold change and dispersion for RNA-seq data with DESeq2. Genome Biol..

[33] Anders S., Huber W. (2010). Differential expression analysis for sequence count data. Genome Biol..

[34] Brunquell J., Morris S., Lu Y., Cheng F., Westerheide S.D. (2016). The genome-wide role of HSF-1 in the regulation of gene expression in *Caenorhabditis elegans*. BMC Genom..

[35] Eden E., Lipson D., Yogev S., Yakhini Z. (2007). Discovering motifs in ranked lists of DNA sequences. PLoS Comput. Biol..

[36] Eden E., Navon R., Steinfeld I., Lipson D., Yakhini Z. (2009). GOrilla: A tool for discovery and visualization of enriched GO terms in ranked gene lists. BMC Bioinform..

[37] Zevian S.C., Yanowitz J.L. (2014). Methodological considerations for heat shock of the nematode *Caenorhabditis elegans*. Methods.

[38] Morita M.T., Tanaka Y., Kodama T.S., Kyogoku Y., Yanagi H., Yura T. (1999). Translational induction of heat shock transcription factor sigma32: Evidence for a built-in RNA thermosensor. Genes Dev..

[39] Athanasiadis A., Rich A., Maas S. (2004). Widespread A-to-I RNA editing of Alu-containing mRNAs in the human transcriptome. PLoS Biol..

