# Supplementary material for: A-to-I RNA Editing Affects lncRNAs Expression after Heat Shock
Source: Genes (Basel). 2018 Dec 13;9(12):627. doi: 10.3390/genes9120627 (PMC6315331; doi:10.3390/genes9120627)
Supplement: Supplementary file 1 [file genes-09-00627-s001.zip › Supplementary figures.pdf]

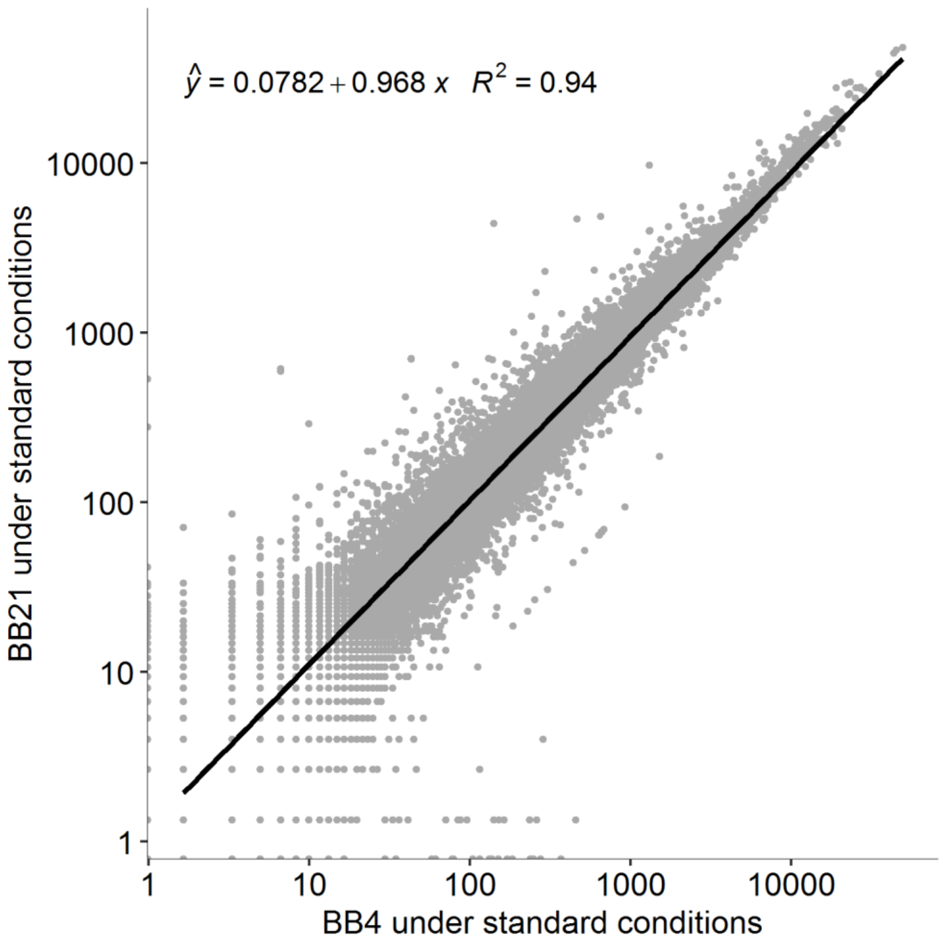

Figure S1. Gene expression levels of the BB4 and BB21 strains are highly correlated. Log scale plots presenting normalized gene counts of the two ADAR mutant worms used in this study, BB21 (*adr-1*(tm668) I; *adr-2*(ok735) III [1]) against BB4 (*adr-1*(gv6) I; *adr-2*(gv42) III [2]), under standard conditions. For each strain one of the biological replicates is presented. Grey dots represent all genes, and the black line is the regression line for all genes.

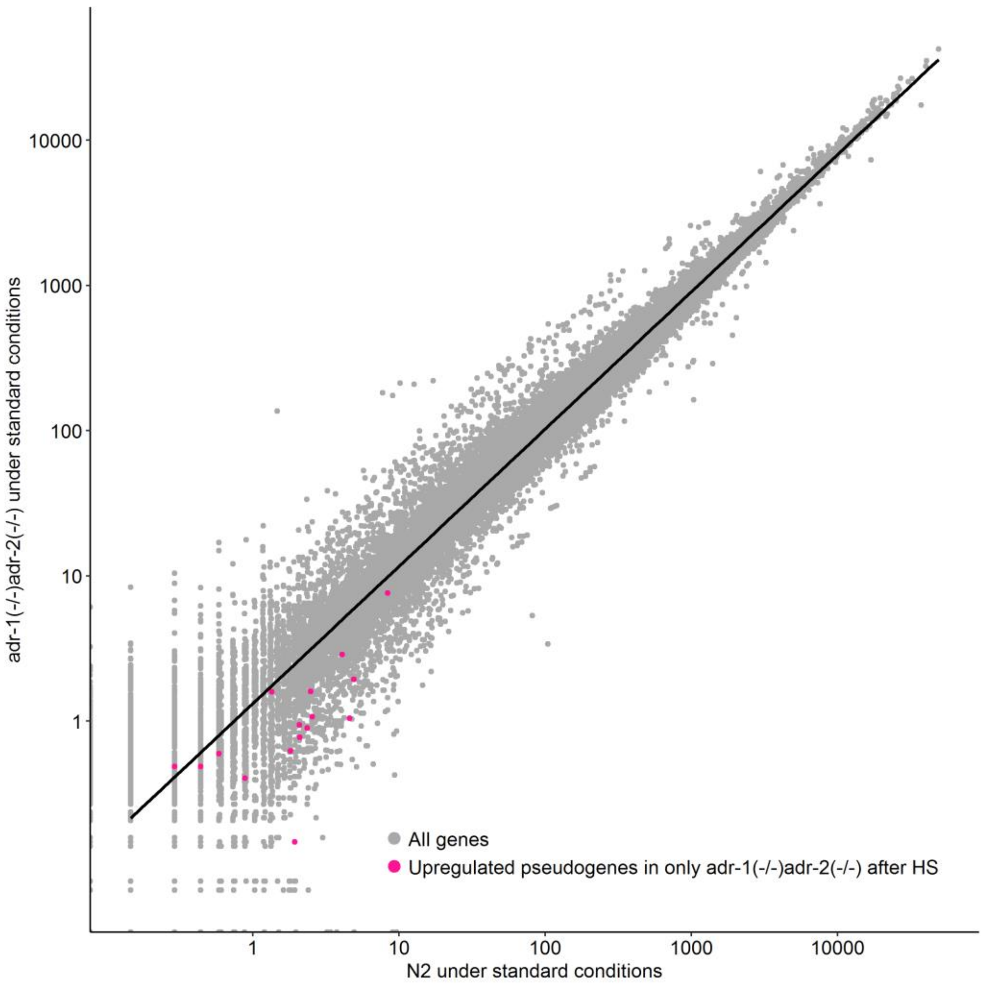

Figure S2. Pseudogenes that are upregulated in the ADAR mutants under heat shock are downregulated in ADAR mutants under standard conditions. Log scale plots presenting normalized gene counts of at least three biological samples in ADAR mutant worms against wildtype (N2) worms, under standard conditions. Grey dots represent all genes (n=23,890), pink dots present pseudogenes and lncRNAs that were upregulated in ADAR mutants under heat shock (n=29), and the black line is the regression line for all genes.

References

1. Hundley, H.A.; Krauchuk, A.A.; Bass, B.L. *C. elegans* C. elegans and *H. sapiens* H. sapiens mRNAs with edited 3′ UTRs are present on polysomes. *RNA* **2008**, *14*, 2050–2060, doi:10.1261/RNA.1165008.
2. Tonkin, L.A.; Saccomanno, L.; Morse, D.P.; Brodigan, T.; Krause, M.; Bass, B.L. RNA editing by ADARs is important for normal behavior in Caenorhabditis elegans. *EMBO J.* **2002**, *21*, 6025–6035.
