# Supplementary material for: A-to-I RNA Editing Affects lncRNAs Expression after Heat Shock
Source: Genes (Basel). 2018 Dec 13;9(12):627. doi: 10.3390/genes9120627 (PMC6315331; doi:10.3390/genes9120627)
Supplement: Supplementary file 1 [file genes-09-00627-s001.zip › Supplementary tables S1-S2.pdf]

Table S1. Enrichment of differentially-expressed genes according to biological process GO term categories

	GO term	Description	P value	P adjusted	[1]Enrichment (N, B, n, b)
DE genes in both N2 and ADAR mutants	GO:0030968	endoplasmic reticulum unfolded protein response	5.02E-17	2.98E-13	12.02 (13581,55,411,20)
	GO:0034976	response to endoplasmic reticulum stress	6.05E-13	1.8E-9	7.78 (13581,85,411,20)
	GO:0006950	response to stress	3.84E-7	7.61E-4	2.06 (13581,850,411,53)
	GO:0030198	extracellular matrix organization	1.35E-6	2.01E-3	6.88 (13581,48,411,10)
	GO:0043062	extracellular structure organization	1.65E-6	1.96E-3	6.74 (13581,49,411,10)
DE genes which are specific to N2	GO:0003824	catalytic activity	1.88E-19	4.76E-16	1.51 (13581,4033,756,338)
	GO:0032559	adenyl ribonucleotide binding	3.42E-19	4.32E-16	2.35 (13581,909,756,119)
	GO:0030554	adenyl nucleotide binding	4.46E-19	3.77E-16	2.34 (13581,912,756,119)
	GO:0043168	anion binding	8.36E-19	5.29E-16	2.04 (13581,1367,756,155)
	GO:0005524	ATP binding	2.29E-18	1.16E-15	2.33 (13581,895,756,116)
	GO:0032555	purine ribonucleotide binding	3.54E-18	1.49E-15	2.16 (13581,1107,756,133)
	GO:0017076	purine nucleotide binding	4.82E-18	1.74E-15	2.15 (13581,1111,756,133)
	GO:0032553	ribonucleotide binding	1.11E-17	3.52E-15	2.13 (13581,1122,756,133)
	GO:0035639	purine ribonucleoside triphosphate binding	1.16E-17	3.25E-15	2.15 (13581,1085,756,130)
	GO:1901265	nucleoside phosphate binding	2.79E-17	7.06E-15	2.04 (13581,1249,756,142)
	GO:0000166	nucleotide binding	2.79E-17	6.42E-15	2.04 (13581,1249,756,142)
	GO:0097367	carbohydrate derivative binding	1.1E-15	2.31E-13	2.01 (13581,1186,756,133)
	GO:0036094	small molecule binding	1.1E-15	2.14E-13	1.92 (13581,1383,756,148)
	GO:0008144	drug binding	2.07E-14	3.75E-12	2.04 (13581,1049,756,119)
	GO:0140096	catalytic activity, acting on a protein	7.64E-14	1.29E-11	1.86 (13581,1359,756,141)
	GO:0016773	phosphotransferase activity, alcohol group as acceptor	3.23E-12	5.12E-10	2.43 (13581,518,756,70)
	GO:0004672	protein kinase activity	5.47E-12	8.15E-10	2.52 (13581,456,756,64)
	GO:0016301	kinase activity	1.51E-11	2.12E-9	2.28 (13581,582,756,74)
	GO:0016772	transferase activity, transferring phosphorus-containing groups	1.52E-9	2.03E-7	2.02 (13581,693,756,78)
	GO:0004715	non-membrane spanning protein tyrosine kinase activity	2.15E-9	2.72E-7	6.12 (13581,47,756,16)
	GO:0004721	phosphoprotein phosphatase activity	8.29E-9	9.99E-7	3.18 (13581,175,756,31)
	GO:0043167	ion binding	8.62E-9	9.91E-7	1.42 (13581,2741,756,216)
	GO:0004725	protein tyrosine phosphatase activity	1.82E-8	2E-6	3.99 (13581,99,756,22)
	GO:0016787	hydrolase activity	2.72E-8	2.87E-6	1.55 (13581,1654,756,143)
	GO:0016791	phosphatase activity	1.5E-7	1.52E-5	2.67 (13581,229,756,34)
	GO:0016740	transferase activity	3.82E-7	3.72E-5	1.51 (13581,1621,756,136)
	GO:0042578	phosphoric ester hydrolase activity	2.4E-6	2.25E-4	2.34 (13581,269,756,35)
	GO:0016788	hydrolase activity, acting on ester bonds	3.77E-6	3.41E-4	1.92 (13581,497,756,53)
	GO:1901363	heterocyclic compound binding	6.18E-6	5.39E-4	1.30 (13581,3011,756,218)
	GO:0097159	organic cyclic compound binding	8.02E-6	6.76E-4	1.30 (13581,3022,756,218)
	GO:0004713	protein tyrosine kinase activity	1.09E-5	8.89E-4	3.32 (13581,92,756,17)

[1]N, total number of genes; B; total number of genes associated with a specific GO term; n, number of differentially expressed genes; b, number of genes in the intersection; Enrichment, (b/n) / (B/N).

Table S2. Enrichment of differentially-expressed genes according to molecular function GO term categories

	GO term	Description	P value	P adjusted	[1]Enrichment (N, B, n, b)
DE genes in both N2 and ADAR mutants	GO:0042302	structural constituent of cuticle	3.01E-36	7.62E-33	9.89 (13581,167,411,50)
	GO:0005198	structural molecule activity	8.43E-21	1.07E-17	4.31 (13581,429,411,56)
	GO:0005201	extracellular matrix structural constituent	2.8E-7	2.36E-4	8.06 (13581,41,411,10)
	GO:0004222	metalloendopeptidase activity	9.63E-6	6.09E-3	4.30 (13581,100,411,13)
DE genes which are specific to N2	GO:0003824	catalytic activity	1.88E-19	4.76E-16	1.51 (13581,4033,756,338)
	GO:0032559	adenyl ribonucleotide binding	3.42E-19	4.32E-16	2.35 (13581,909,756,119)
	GO:0030554	adenyl nucleotide binding	4.46E-19	3.77E-16	2.34 (13581,912,756,119)
	GO:0043168	anion binding	8.36E-19	5.29E-16	2.04 (13581,1367,756,155)
	GO:0005524	ATP binding	2.29E-18	1.16E-15	2.33 (13581,895,756,116)
	GO:0032555	purine ribonucleotide binding	3.54E-18	1.49E-15	2.16 (13581,1107,756,133)
	GO:0017076	purine nucleotide binding	4.82E-18	1.74E-15	2.15 (13581,1111,756,133)
	GO:0032553	ribonucleotide binding	1.11E-17	3.52E-15	2.13 (13581,1122,756,133)
	GO:0035639	purine ribonucleoside triphosphate binding	1.16E-17	3.25E-15	2.15 (13581,1085,756,130)
	GO:1901265	nucleoside phosphate binding	2.79E-17	7.06E-15	2.04 (13581,1249,756,142)
	GO:0000166	nucleotide binding	2.79E-17	6.42E-15	2.04 (13581,1249,756,142)
	GO:0097367	carbohydrate derivative binding	1.1E-15	2.31E-13	2.01 (13581,1186,756,133)
	GO:0036094	small molecule binding	1.1E-15	2.14E-13	1.92 (13581,1383,756,148)
	GO:0008144	drug binding	2.07E-14	3.75E-12	2.04 (13581,1049,756,119)
	GO:0140096	catalytic activity, acting on a protein	7.64E-14	1.29E-11	1.86 (13581,1359,756,141)
	GO:0016773	phosphotransferase activity, alcohol group as acceptor	3.23E-12	5.12E-10	2.43 (13581,518,756,70)
	GO:0004672	protein kinase activity	5.47E-12	8.15E-10	2.52 (13581,456,756,64)
	GO:0016301	kinase activity	1.51E-11	2.12E-9	2.28 (13581,582,756,74)
	GO:0016772	transferase activity, transferring phosphorus-containing groups	1.52E-9	2.03E-7	2.02 (13581,693,756,78)
	GO:0004715	non-membrane spanning protein tyrosine kinase activity	2.15E-9	2.72E-7	6.12 (13581,47,756,16)
	GO:0004721	phosphoprotein phosphatase activity	8.29E-9	9.99E-7	3.18 (13581,175,756,31)
	GO:0043167	ion binding	8.62E-9	9.91E-7	1.42 (13581,2741,756,216)
	GO:0004725	protein tyrosine phosphatase activity	1.82E-8	2E-6	3.99 (13581,99,756,22)
	GO:0016787	hydrolase activity	2.72E-8	2.87E-6	1.55 (13581,1654,756,143)
	GO:0016791	phosphatase activity	1.5E-7	1.52E-5	2.67 (13581,229,756,34)
	GO:0016740	transferase activity	3.82E-7	3.72E-5	1.51 (13581,1621,756,136)
	GO:0042578	phosphoric ester hydrolase activity	2.4E-6	2.25E-4	2.34 (13581,269,756,35)
	GO:0016788	hydrolase activity, acting on ester bonds	3.77E-6	3.41E-4	1.92 (13581,497,756,53)
	GO:1901363	heterocyclic compound binding	6.18E-6	5.39E-4	1.30 (13581,3011,756,218)
	GO:0097159	organic cyclic compound binding	8.02E-6	6.76E-4	1.30 (13581,3022,756,218)
	GO:0004713	protein tyrosine kinase activity	1.09E-5	8.89E-4	3.32 (13581,92,756,17)
DE genes which are specific to ADAR mutant	GO:0098772	molecular function regulator	2.98E-6	7.56E-3	2.04 (13581,396,772,46)
	GO:0004867	serine-type endopeptidase inhibitor activity	5.28E-6	6.68E-3	4.69 (13581,45,772,12)
	GO:0004866	endopeptidase inhibitor activity	1.39E-5	8.82E-3	4.01 (13581,57,772,13)

[1]N, total number of genes; B; total number of genes associated with a specific GO term; n, number of differentially expressed genes; b, number of genes in the intersection; Enrichment, (b/n) / (B/N).
